# Colorectal Cancer Study with Nanostructured Sensors: Tumor Marker Screening of Patient Biopsies

**DOI:** 10.3390/nano10040606

**Published:** 2020-03-26

**Authors:** Michele Astolfi, Giorgio Rispoli, Gabriele Anania, Veronica Nevoso, Elena Artioli, Nicolò Landini, Mascia Benedusi, Elisabetta Melloni, Paola Secchiero, Veronica Tisato, Giulia Zonta, Cesare Malagù

**Affiliations:** 1 Department of Physics and Earth Sciences, University of Ferrara, Via Saragat 1/C, 44122 Ferrara, Italy; stlmhl@unife.it (M.A.); lndncl@unife.it (N.L.); zntgli@unife.it (G.Z.); 2 SCENT S.r.l (SME company), Via Quadrifoglio 11, 44124 Ferrara, Italy; 3 Department of Biomedical and Specialist Surgical Sciences, University of Ferrara, Via Luigi Borsari 46, 44121 Ferrara, Italy; rsg@unife.it (G.R.); bndmsc@unife.it (M.B.); mlllbt@unife.it (E.M.); 4 Department of Morphology, Surgery and Experimental Medicine, University of Ferrara, Via Luigi Borsari 46, 44121 Ferrara, Italy; ang@unife.it (G.A.); veronica.nevoso@student.unife.it (V.N.); elena01.artioli@student.unife.it (E.A.); paola.secchiero@unife.it (P.S.); veronica.tisato@unife.it (V.T.)

**Keywords:** nanostructured sensors, tumor markers, colorectal cancer, human cancer biopsies, chemoresistivity, volatile organic compounds

## Abstract

Despite the great progress in screening techniques and medical treatments, colorectal cancer remains one of the most widespread cancers in both sexes, with a high death rate. In this work, the volatile compounds released from human colon cancer tissues were detected by a set of four different chemoresistive sensors, made with a nanostructured powder of metal-oxide materials, inserted into an innovative patented device. The sensor responses to the exhalation of a primary cancer sample and of a healthy sample (both of the same weight, collected during colorectal surgery from the intestine of the same patient) were statistically analyzed. The sensors gave reversible, reproducible, and fast responses for at least one year of continuous use, making them quite superior in respect to the existing diagnostic methods. Preliminary results obtained using principal component analysis of the sensor responses to samples removed from 13 patients indicate that the nanostructured sensors employed in this study were able to distinguish between healthy and tumor tissue samples with coherent responses (the discrimination power of the most sensitive sensor was about 17%), highlighting a strong potential for clinical practice.

## 1. Introduction

Colorectal cancer (CRC) is the third most common cancer worldwide with an increasing trend with age, and second in causing mortality in both sexes [1]. Considering the increasing incidence of this disease [1], its mortality and lethality, the impossibility of preventing its insurgence solely by changing the patient’s habits, and the lack of symptoms when it is reaching an advanced phase, screening programs are of paramount importance. Equally important is the follow-up of patients that have undergone surgery and/or chemo-radiotherapy, to assess the therapy’s efficacy. Therefore, it is necessary to develop new devices able to detect known tumoral markers with higher sensitivity, or to detect new ones. Since tumor growth is associated with modifications in cell metabolism, in recent years, the scientific community has focused on detecting molecules discharged by this metabolism in blood, feces, urine, breath, etc., or in samples surgically removed from patients. The molecules so far identified are:

- Vascular endothelial growth factors;
- Wastes from circulating tumor cells;
- Molecules produced by the cellular metabolism;
- Products of lipid membrane peroxidation, known as volatile organic compounds (VOCs) [2,3,4].

In particular, many CRC tumors are often associated with specific VOC patterns (such as benzene, alkanes, aldehydes or their derivatives) that can be detected in the body at different concentrations in healthy people [4]. Therefore, VOC detection appears highly promising as a possible marker of CRC, and more generally of other neoplastic diseases.

A project aiming to detect VOCs released by colorectal neoplasms started in 2014, with the development of a device based on an array of chemoresistive nanostructured sensors, optimized to detect these gases [5]. This device, built by the research team of a startup named SCENT S.r.l., is not invasive and is fast responding; therefore, it could be employed in the future with existing methods to improve the diagnostic ability to detect CRC at the screening stage. The instrument could also be employed in the follow-up phase of patients who have already undergone surgery and/or radiotherapy and/or chemotherapy, to assess the therapies’ efficacy. The ultimate goal is to significantly decrease the number of false negatives or positives, which still affect other commonly employed pre-screening tests (such as fecal immunochemical techniques and colonoscopy).

### 1.1. Metal-Oxide Chemoresistive Sensors

**Figure 1:**
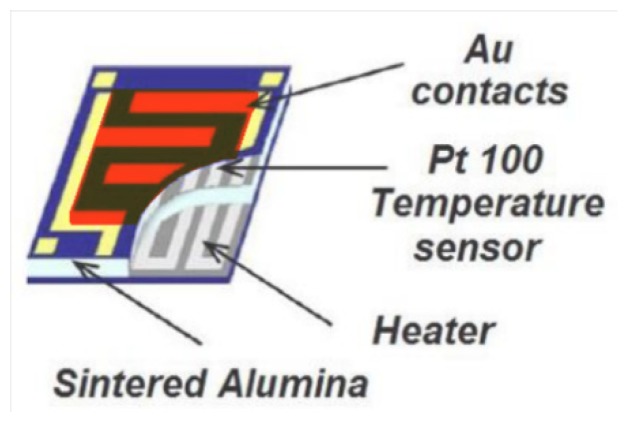
Top view of a sensor. The light blue central layer is the alumina substrate; the yellow lines are the gold contacts employed to connect the sensor to the external circuits; the grey coil at the bottom face of the substrate is the heater; the red area is the active material, distributed between the gold contacts.

MOX(Metal-Oxide) sensors are based on semiconducting metal-oxide material that are able to detect chemicals up in the range of tenth parts per billions, despite their rapid response. The working principle relies on the conductance change of the sensitive material induced by the reversible adsorption of ionized gas particles on the sensor surface [6,7,8]. The sensors are typically made from three components: a substrate, an active (sensitive) material, and a heater. The latter is in general necessary because MOX sensors are active at a specific working temperature. The substrate is an insulating layer, made in this work of sintered alumina, hosting on top the active material with interdigitated gold contacts, and on the bottom, the heater (a typical sensor is sketched in Figure 1) [9]. The active material is made of semiconducting metal-oxide nanoparticles (having a mean size between 50 and 200 nm), which was chemically transformed here in a viscose paste that was distributed on the top of the insulating substrate using a lithographic technique. The heater is usually a metal coil (in this work made of platinum) whose temperature is precisely set by controlling the current flowing through it. 

### 1.2. Prototypes

A first prototype, SCENT A1, has been used to date as a CRC screening method by measuring feces samples, and it gave significant and reproducible results [5]. Since neoplasms discharge a substantial amount of specific VOCs in the blood stream [9,10], being lesions that are highly vascularized, a second prototype (named SCENT B1) was specifically developed to detect blood [9] and human tissue VOC exhalations (this study). The statistical analysis of SCENT B1 sensor responses to blood samples allowed reliable discrimination between healthy and tumor-affected subjects, and it was even able to identify tumors at evolutionary stage [9]. On this basis, SCENT B1 was then used in the present work to analyze VOCs exhaled by biopsies explanted from patients who underwent colon and/or rectal resections. The main advantage of this approach lies in measuring directly the VOCs exhaled by tumor tissue, and not altered by other chemical reactions that take place inside the human body.

## 2. Materials and Methods

### 2.1. SCENT B1 Device

The patented device SCENT B1 employed in this work [11] has been widely described in a previous publication of the team [9]. In detail, the sensors used were:

TiTaV, based on titanium, tantalum and vanadium oxides;

STN, based on tin, titanium, and niobium oxides;

ST 25 + 1%Au, based on tin oxides and titanium (25%) and gold (1%);

W11, based on tungsten oxide.

All sensors were fired to a temperature of 650 °C and worked at a temperature set to 450 °C to activate the semiconductor layer, allowing chemoresistivity to occur. As shown in the literature [9,10,12], this high temperature does not affect substantially the analyte’s composition nor the laminarity of the flux entering the sensing chambers. The sensors listed above were chosen according to their capability of recognizing tumor markers, which was assessed in previous research by using standard pure gases, feces [5], and blood [9].

**Figure 2:**
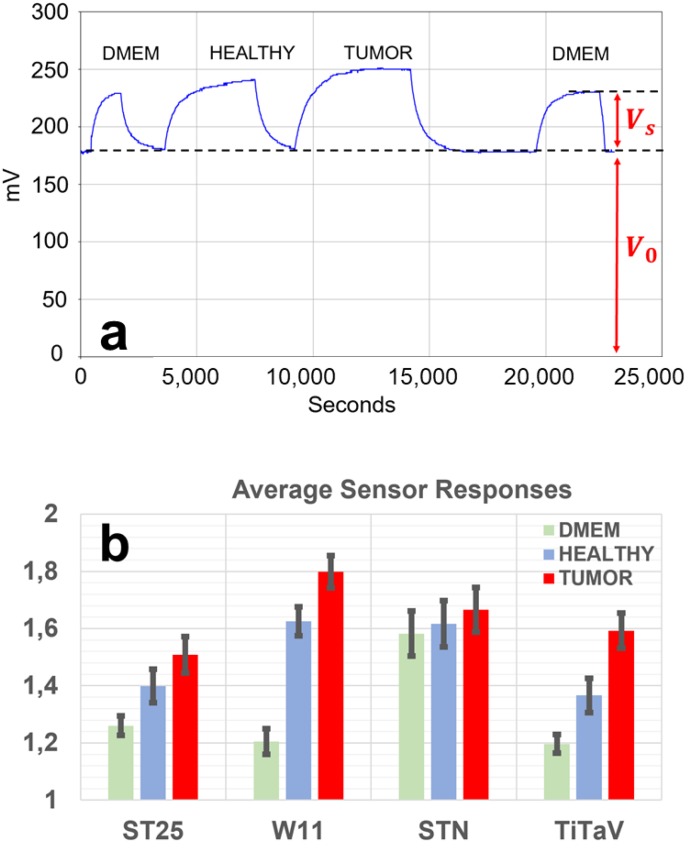
(**a**) Experimental protocol and time-course of a representative sensor response. The response amplitude of the sensor with the highest discrimination power, TiTaV, (titanium, tantalum, and vanadium oxide) is plotted vs. time; the sensor was exposed to the following sample sequence: clean air/DMEM/clean air/healthy sample/clean air/tumor sample/clean air/DMEM/clean air. (**b**) Average sensor responses. Bar graph of the average ratio (*n* = 13) between the sensor response to a flux of air containing the gasses exhaled by DMEM (green bars), healthy tissue (light blue), and tumor tissue (red); and its response to a flux of clean air (baseline); error bars represent the standard error. The sensors employed were ST25 (tin and titanium oxide), W11 (tungsten oxide), STN (tin, titanium, and niobium oxide), and TiTaV.

To make the results independent of the voltage baseline at which each sensor worked, the response R(t) was computed as:

(1) $$R\left(t\right)=\frac{V_{sens}\left(t\right)}{V_{0}\left(t\right)}$$

where $V_{sens}$ is the average voltage output of the operational amplifier connected to the sensor, used in inverting configuration, in the presence of the sample, and $V_{0}$ is the average voltage measured in its absence (i.e., when the sensor is exposed to a flux of clear air; Figure 2a).

### 2.2. Patient Recruitment and Sample Collection

Colorectal tissue samples and related clinical data were obtained upon written consent from patients who underwent open or laparoscopic surgery for CRC in the Hospital of Cona, Ferrara, Italy. All patients were more than eighteen years old, of both sexes, and did not have any radiotherapy and chemotherapy before surgery; minors and pregnant women were excluded from the study; all tumors tested were malignant, and some had already metastasized to liver (multicentric bilobar replications).

The tumor and the healthy samples were cut out from the same intestine piece, trying to collect as much tumor mass as possible (since the healthy mass around the tumor was always much larger than the tumor itself), to maximize the sensor response and consequently the signal-to-noise ratio.

The time elapsed between the surgical removal of the sample and the SCENT B1 tests was kept as brief as possible. However, an average of 45 min elapsed between devascularization and sample explant, which was immediately immersed in culture medium. The latter, named DMEM throughout the paper, was Dulbecco’s modified Eagle’s medium high glucose (DMEM, Lonza^®^, Milan, Italy) supplemented with 10% fetal bovine serum (Euro Clone, Milan, Italy), l-glutamine at 1% (Lonza), and the antibiotics penicillin (100 U/mL) and streptomycin (100 μg/mL) at 1% (Lonza). Moreover, about two additional hours elapsed between the transport and sample preparation; therefore, a significant number of cells were lost during these three hours, but this was roughly the same for both healthy and tumor cells.

### 2.3. Sample Handling

Careful dissection was employed to isolate as much as possible of the cancer and the healthy tissue, both trimmed to have the same mass, which was assessed with mg accuracy (average sample mass 0.282±0.052 gr; range: 0.0432 gr–0.676 gr; *n*= 13). The samples were then carefully washed with phosphate buffered saline (PBS, 1X, Lonza) supplemented with penicillin (100 U/mL), streptomycin (100 μg/mL), and amphotericin B (250 μg/mL) at 1% (Lonza), to eliminate as much blood, feces residue, and other organic debris as possible, and to further sterilize the samples. The cleanliness of the latter was routinely checked by viewing them on a screen connected to a high-sensitivity digital CMOS camera, using a 2.3 megapixel CMOS sensor (C11440-36U, Hamamatsu Photonics, Tokyo, Japan) coupled to a microscope (TE 300, Nikon, Tokyo, Japan) with high magnification (Figure S1). Further details of sample handling are described in the supplementary materials. 

### 2.4. Ethics Approval and Informed Consent

The trial protocol and the informed consent form were presented, accepted, and retrospectively registered by the Ethical Committee of the District of Ferrara, with trial number 170484, on 13 July 2017.

## 3. Results

First, we analyzed the average ratio of the response of each sensor to VOCs exhaled by a sample and the response of the sensor to a flux of clean air, using Formula 1, (Figure 2; see Section 2). The samples were DMEM (see Section 2), a healthy tissue sample, and its tumor tissue counterpart (i.e., both taken from the same surgical sample); all sensors were simultaneously exposed to the VOCs exhaled by the samples. The sensors gave responses that were reversible (the baseline was fully recovered following a sample measurement), reproducible (an example is shown in Figure 2a, where the response of a sensor to DMEM had similar amplitude to the one recorded after the healthy and tumor sample measurement), and quite fast (the sensor response reached a steady state typically in less than half an hour) for at least one year of continuous use.

The sensor discrimination power to discern between healthy and tumor samples, given by:

(2) $$discrimination\;power=\left(\frac{T}{H}-1\right)\times 100$$

resulted in: ~7.8% for ST25, ~10.7% for W11, ~3% for STN, and ~16.6% for TiTaV (*H* is the average ratio of each sensor response to a healthy sample; i.e., the light blue bar height of Figure 2b; *T* the tumor one; i.e., the red bars). Given the poor selectivity exhibited by the STN sensor for the sample type considered here, its responses were therefore not considered in the following statistical analysis.

To better assess the discrimination power of the set of three sensors (ST25, W11, and TiTaV), their responses were further analysed with principal component analysis (PCA). The three eigenvectors of the covariance matrix (PC1, PC2, PC3), calculated from the three-dimensional plot of the sensor responses, were plotted one vs. the other (PC1 vs. PC2, PC2 vs. PC3, and PC1 vs. PC3) to construct the two-dimensional dispersion graphs.

**Figure 3:**
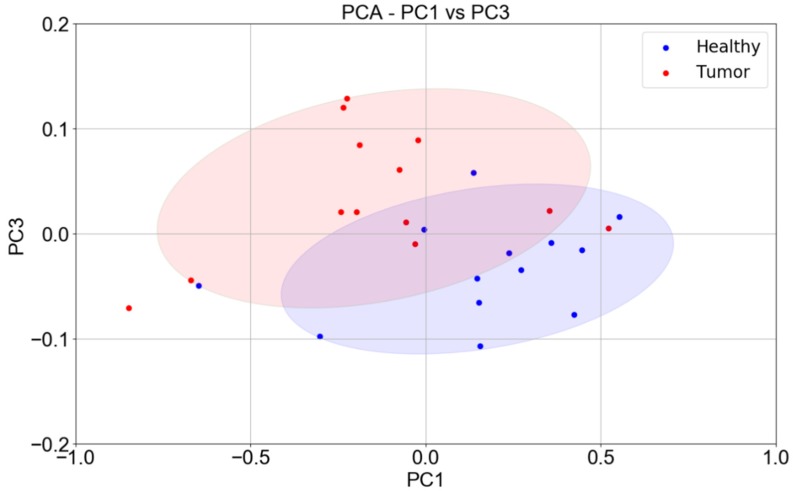
PCA (principal component analysis) of sensor responses—PC1 vs. PC3. PCA score plot (blue points: healthy tissues; red points: tumor tissue) constructed with the responses (*n* = 13) of the ST25, W11, and TiTaV sensors only.

The plot of PC1 vs. PC3 was the most discriminating one, and it contained the largest share of the total variance of the covariance matrix (about 90.3% of information, Figure 3). The confidence ellipses resulting from the score plot of PC1 vs. PC3 were well separated, further indicating that the strategy employed to develop SCENT B1 to discriminate between healthy tissues and cancerous ones was promising.

To further explore the discriminating power of SCENT B1, we also considered the tumor grade index, which quantifies the differentiation grade of tumor cells [13], therefore expressing the tumor’s malignancy. In principle, a high-grade tumor, growing faster than a low-grade one, should have a higher metabolism and consequently a larger production of known VOCs and/or different ones because of cell indifferentiation. Therefore, it is expected that high-grade tumors generate sensor responses different to the low-grade ones. As a control, we plotted PC1 vs. PC3 of the healthy tissue only to distinguish the healthy counterparts of the high-grade tumors from the low-grade ones.

**Figure 4:**
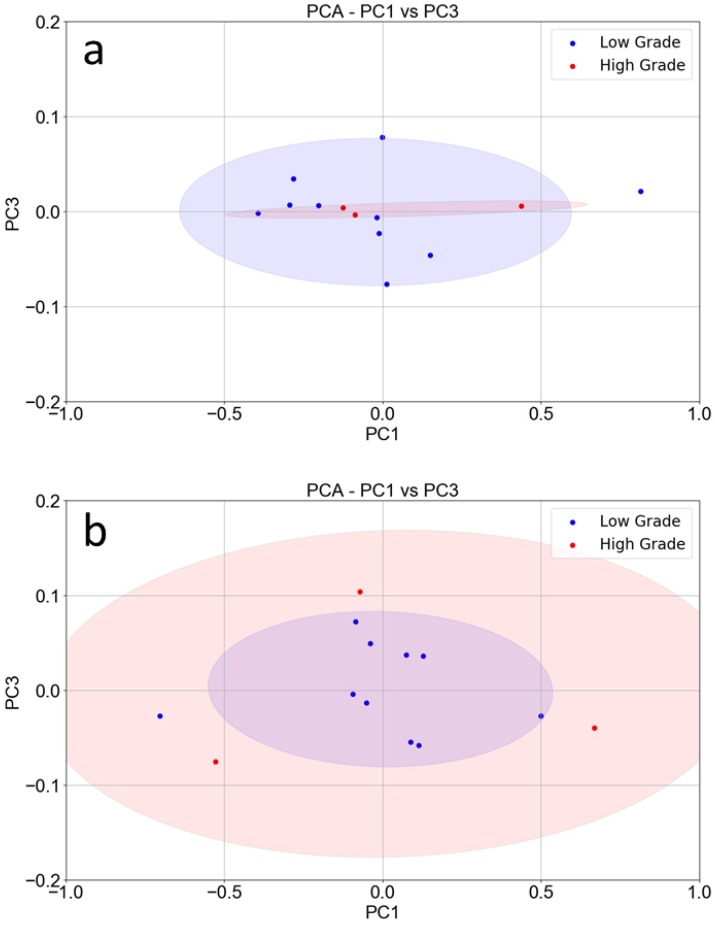
PCA of sensor responses related to tumor grade index: (**a**) PC1 vs. PC3 score plot constructed with the responses (*n* = 13) of the ST25, W11, and TiTaV sensors to the healthy sample counterparts of the low-grade tumors (blue points) and to the high-grade ones (red points). (**b**) PC1 vs. PC3 score plot constructed with the responses (*n* = 13) of the same sensors to the low-grade tumor samples (blue points) and to the high-grade ones (red points).

As expected, the points relative to the healthy tissue counterparts of the high-grade tumors (red) were completely confused with the low-grade ones (blue; Figure 4a). Indeed, a healthy sample removed from a certain intestine area is believed to produce the same type and amount of VOCs independent of the presence or not of a tumor (of whatsoever grade) in another area. The PCA performed on tumor tissue responses (Figure 4b) produced instead two concentric confidence ellipses, where all the points (but one) relative to the low-grade tumor samples (blue) were concentrated in an ellipse (violet), while all the points relative to the high-grade tumor samples (red) were outside of the violet ellipse and inside the larger one (pink).

## 4. Discussion

As expected, the sensor response amplitudes were larger in the presence of the tumor tissue in respect of the same mass of healthy tissue. Indeed, tumor cells exhale more VOCs than the healthy ones because of a higher growth rate and, consequently, an accelerated metabolism of the former in respect to the latter.

Despite the poor selectivity and discrimination power exhibited by the STN sensor for the sample types considered here, the other three sensors (ST25, W11, and mainly TiTaV) were instead able to distinguish healthy from tumor samples, with a different selectivity (Figure 2b): This gives a key rationale to design sensors with better and better sensitivity and discriminating power.

In general, the sensitivity (TPR) and specificity (TNR) of the performance of a binary classification test are defined as:

(3) $$TPR=\frac{TP}{P}\;TNR=\frac{TN}{N}$$

where P is the number of subjects of the total, $N_{t}$, that are positive to the test, $N_{t}-P=N$ are the negative ones (control subjects), and TP and TN are the number of true positives and true negatives (to the test), respectively. This approach cannot be applied here, because the population of $N_{t}$ subjects are all positive, i.e., affected by CRC tumors, and from these patients, an intestine piece was surgically removed from which both a tumor and healthy sample were isolated. Therefore, strict interpretation of TPR and TNR is meaningless here, since no measures were performed on healthy subjects. It is still possible to give an estimate of these parameters, reinterpreting them on the basis of the PCA analysis (Figure 3) if P is considered the number of tumor samples and $N_{t}-P=N$ the number of healthy control samples. On the basis of Figure 3, excluding the points outside the confidence ellipses and considering the points inside the common area as false positives and false negatives, the sensitivity and specificity results are TPR=66.6% and TNR=69.2%. These values must be considered with caution, since PCA is a qualitative method to classify data and, in general, an accurate evaluation of sensitivity and specificity requires much larger samples than the one presented here.

These results were quite encouraging considering the strong limits of the measurements performed: First of all, the tumor mass extracted after surgery was limited because of the necessity to perform a histological analysis for cancer staging. Moreover, the samples were composed of different kinds of healthy connective tissue and intestine cells—fat, red, and white blood cells, floral bacteria, feces residues, and neoplastic cells—possibly in different development stages, making it impossible even to distinguish healthy samples from tumor ones, despite the high quality of their microscope images (Figure S1, Supplementary Materials). Another limitation is due to the possible tissue damage occurring during the elapsed time between surgery and the experimental session (see Section 2).

## 5. Conclusions

This paper is intended as a feasibility study to find the most suitable sensors and statistical analysis to discriminate as well as possible between the VOCs exhaled by tumors and healthy samples. The set of nanostructured chemoresistive sensors—ST25, W11, and TiTaV—employed in this study proved to be capable of discriminating between healthy and tumor-affected biopsies, and even able to distinguish their grade of differentiation. These encouraging results will be followed by a wider study, which will involve many more cases and broaden the ongoing collaboration with pathologists. The final goal is the future development of pre- and post-screening devices that could add to existing methods, such as conventional colonoscopy, to improve tumor detection.

## 6. Patents

The Scent B1 device is patented in Italy with patent number: 102015000057717 [11].

## Supplementary Materials

The following are available online at https://www.mdpi.com/2079-4991/10/4/606/s1. Figure S1: Optical microscope images from colorectal tissue.
