# Supplementary material for: Colorectal Cancer Study with Nanostructured Sensors: Tumor Marker Screening of Patient Biopsies
Source: Nanomaterials (Basel). 2020 Mar 26;10(4):606. doi: 10.3390/nano10040606 (PMC7221558; doi:10.3390/nano10040606)
Supplement: Supplementary file 1 [file nanomaterials-10-00606-s001.pdf]

SUPPLEMENTARY MATERIAL

Material and methods

To maximize the sample surface emitting gas and to have them thoughtfully soaked in culture medium at the same time, each sample was gently triturated as described below, and equally distributed in three petri dishes that were inserted in the sample chamber of SCENT B1, as described in (Landini, et al. 2018). The two samples (an exemplificative microphotograph of them is shown in Fig. S1) were then transferred from PBS into a teflon support having two separate trays (one for the healthy and one for the tumor sample) containing 1.8 ml of DMEM. By using a sterilized scalpel and a pair of tweezer, the healthy and the tumor samples were shred under a fume hood, and a further fine trituration was performed with a 1 ml pipette, with its plastic tip cut obliquely with a razor blade. A 0.6 ml aliquot of a triturated sample was pipetted into a petri dish containing 0.9 ml of DMEM, so to have three petri dishes each containing 1.5 ml of healthy sample and three containing the tumor one. In parallel, other three petri dishes each containing 1.5 ml of DMEM only were prepared (as a control); the 9 petri dishes were kept in a dedicated incubator (at a temperature of 37°C with 95% air and 5% CO_2) for the time strictly necessary to perform the measurements with SCENT B1.

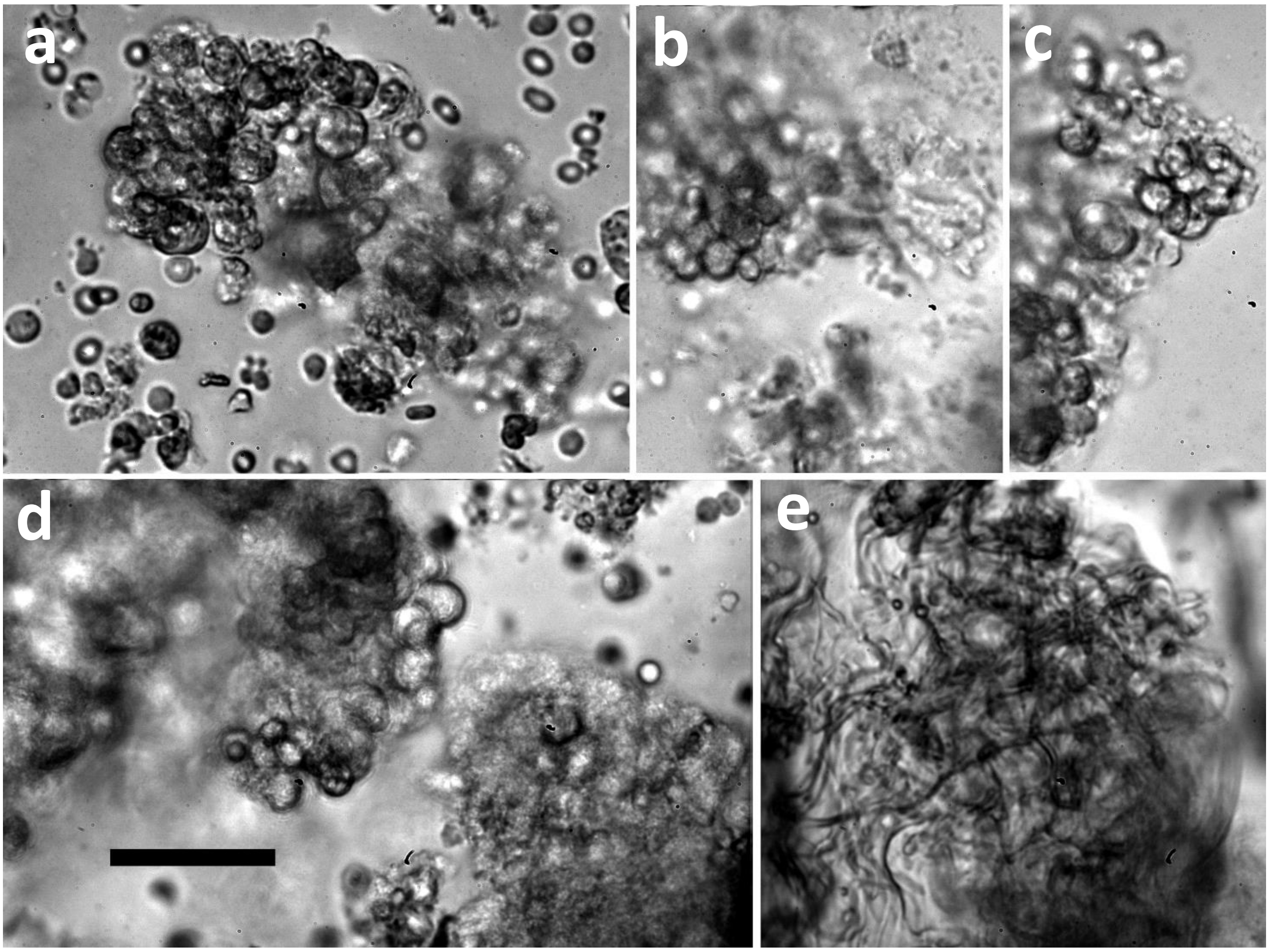

Figure S1: *Optical microscope images from colorectal tissue.* Bright field images of tumor samples (*a* and *d*) and of healthy ones (*b*, *c*, *e*); opalescent bubbles are composed by fat, while concave isolated cells are red blood cells. Black scale bar in *d* is 50 µm long and applies to all images.
